# Regional and seasonal drivers of metals and PAHs concentrations in road dust and their health implications in the Czech Republic

**DOI:** 10.1016/j.heliyon.2024.e40725

**Published:** 2024-11-26

**Authors:** Radim Seibert, Bohumil Kotlík, Helena Kazmarová, Václav Dombek, Vladimíra Volná, Daniel Hladký, Blanka Krejčí

**Affiliations:** a Czech Hydrometeorological Institute, K Myslivně 3/2182, 708 00, Ostrava-Poruba, Czech Republic; b The National Institute of Public Health, Šrobárova 49/48, 100 00 Prague 10, Czech Republic; c VSB - Technical University of Ostrava, 17. listopadu 2172/15, 708 00, Ostrava-Poruba, Czech Republic

**Keywords:** Road dust, PAH, Metals, Resuspension, Health risk, PMF

## Abstract

While car exhaust emissions in the EU are clearly decreasing, the future of non-exhaust emissions looks more pessimistic. The relative importance of the latter is thus expected to increase in terms of air quality and human health. The aim of the study was to assess regional and seasonal differences in the chemical composition of road dust across the Czech Republic and the health impact of its resuspension, with special respect to polycyclic aromatic hydrocarbons and metals. The road dust samples across all regions and seasons were collected. Based on subsequent laboratory and statistical processing, the spatiotemporal distribution of elements and PAHs was evaluated. Next, the contribution of road dust resuspension to air concentrations was estimated and related health impacts were assessed. A significant regional and seasonal variations in PAHs and metals were discovered. Air quality, leading to atmospheric deposition, was the most important factor contributing to these variations. In contrast, road traffic intensity played only a minor role in influencing the concentrations of metals and PAHs in road dust. Exposure to the PM_10_ fraction of road dust led to an increase in premature mortality, postneonatal infant mortality, and the prevalence, occurrence, and incidence of bronchitis by several percent. It also significantly raises the annual rate of emergency respiratory hospitalizations and the number of days per year using bronchodilators. Exposure to PAHs and heavy metals in road dust causes cancer incidence on the order of a few cases per 10 million people. Air quality protection measures that lead to a decrease in atmospheric deposition rates are required for the effective reduction of health risks associated with particle resuspension by traffic.

## Highlights

• Elemental and PAHs concentrations in road dust varied regionally and seasonally.
• Traffic intensity had only a minor effect on the chemical composition of road dust.
• Atmospheric deposition significantly affected the metals and PAHs content in road dust.
• The adverse effects of PM_10_ in road dust were more serious than those of metals and PAHs.
• Improving air quality is essential to mitigate health risks from road dust resuspension.

## 1 Introduction

Road dust constitutes a significant portion of traffic-related PM emissions [1,2]. Its chemical composition can be influenced by various factors, including automobile emissions, atmospheric deposition, road surface quality, and maintenance, among others [3]. With the anticipated future reduction of combustion engines in personal transportation, a substantial decrease in traffic exhaust emissions is expected in the EU, including the Czech Republic. However, in contrast to exhaust emissions, it is likely that road dust resuspension and emissions from brake, tire, and road wear will not change significantly, as the similar road surfaces, tires, and brakes will continue to be used regardless of the type of vehicle propulsion. Some authors expect stagnation in brake wear emissions due to the higher weight of electric vehicles [4], while others anticipate a decrease because of the adoption of regenerative braking systems [5]. The emissions from brake, tire, and road wear can influence the chemical composition of road dust [6] and, consequently, the toxicological effects of particles resuspended by traffic [7]. The mentioned uncertainties regarding future brake wear emission levels affect the quality of road dust but there is no evidence of significant impact on total non-exhaust emission rate. The proportion of total non-exhaust traffic emissions gradually increase [8,9], and road dust is becoming increasingly important for both air quality and public health protection.

Atmospheric aerosol particulate matter (PM) consists of a complex mixture of chemical substances with various properties. The interpretation of findings regarding exposure and related health risks is complicated by the fact that the potential of PM to affect health depends on the chemical and physical parameters of the particles. Different characteristics are relevant for different types of health impacts. Health effects of PM are influenced by the adsorption of various pollutants on the particle surface. In the case of road dust emissions, PM is a known carrier, especially for metals and polycyclic aromatic hydrocarbons, which are harmful chemical substances [10,11]. The significant toxicological effect of polycyclic aromatic hydrocarbons in road dust was evaluated also in the previous study assessing the situation in Poland [12], which is a neighboring country to the Czech Republic. However, health effects related to resuspended road dust are typically assessed without taking into account the presence of these pollutants. The health risk quantification usually assumes that PM itself, as well as the mentioned ambient air pollutant mixture bound on the particle surface, has been listed as a proven human carcinogen in category 1 by The International Agency for Research on Cancer (IARC) of the World Health Organization (WHO) since 2013, contributing to the development of lung cancer.

The aim of the scope was to quantify the content of PAHs and heavy metals in road dust across the Czech Republic and assess their impact on human health. For these purposes, road dust monitoring was carried out and the contribution of road dust, heavy metals, and PAHs resuspended by traffic to PM_10_ ambient air concentrations were determined based on the PMF source apportionment. Based on these information, the health risk associated with road dust resuspension, including the contributions of metals and PAHs, was estimated.

## 2 Methods

### 2.1 Sampling

**Fig. 1:**
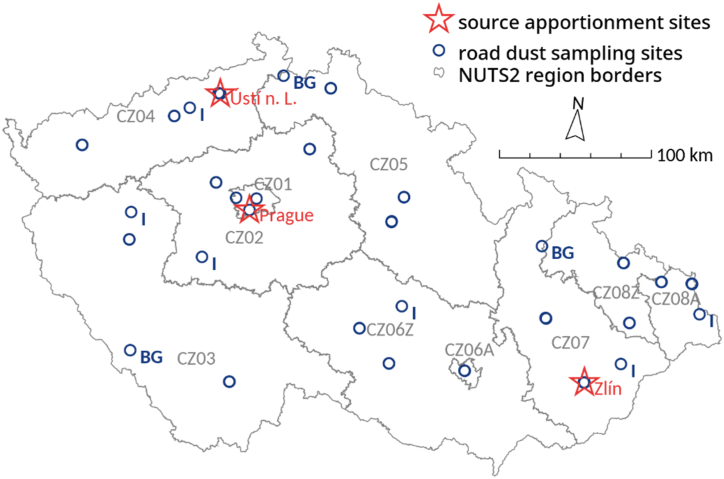
Sampling sites; BG: roads at mountain background sites; I: roads near large industrial facilities; unmarked: city sites.

Sampling for the air pollution source apportionment was conducted in Prague (lat. 50.0065° N, lon. 14.4483° E), Ústí nad Labem (lat. 50.6630° N, lon. 14.0316° E), and Zlín (lat. 49.2217° N, lon. 17.6599° E). These cities serve as the capitals of regions in the Czech republic (Fig. 1), all characterized by heavy road traffic. Road dust samples were collected at 50 road profiles, evenly distributed across ten air quality zones in the Czech Republic, designated CZ01 to CZ08Z, to investigate regional variations in road dust chemical composition (Fig. 1).

For the source apportionment in aforementioned three cities, atmospheric PM_10_ aerosol sampling on filters was conducted using automatic SEQ samplers, Sven Leckel – Ingenieurbüro GmbH, Germany, with a controlled flow of 2.3 m^3^ h^−1^ and an accuracy of 2 %. The samples were collected from 6th October to December 5, 2020 and 1st June to July 31, 2021. The aim of the seasonal sampling was incorporating diferent meteorological conditions and air pollution sources activity in diferent seasons to the source apportionment. As shown in Table S1, the total number of samples valid for PMF varied between 106 and 120 among the source apportionment sites depicted in Fig. 1.

Road dust from the road surface was sampled in selected cities in all seasons. As the study was focused on health impacts, the most of the samples were collected in cities. The sampling sites in cities were placed in pairs. Both sites of the pair were placed within maximum distance of several hundreds meters. The goal of such a distribution was to ensure that the samples were affected by the atmospheric deposition of emissions from the same air pollution sources. The difference between the sites within each pair was the road traffic intensity. One site from the pair was on the road with the daily intensity ≥7500 and the second with <7500 vehicles per day. A limited number of sampling sites were placed near the industrial PM sources to evaluate their impact on road dust parameters. Additionally, roads in three background sites in the mountains near the Czech republic border were sampled to survey road dust parameters in the areas where traffic is the only significant local pollution source (“BG” mark in Fig. 1). In total, 197 samples were collected and subsequently processed (Table S2).

The objective of road dust sampling was to assess the variation in relative chemical composition. The road dust emission rate was not a concern, as the methodology for such an assessment [13] is well established and has been routinely used for a long time. Following this purpose, continuous, 50–200 m long road sections were sampled in each sampling profile, with an active sampling zone of 0–0.5 m from the roadside. Repeating of seasonal sampling in the same locations was ensured by GPS recording. The raw road dust samples were collected using Makita DVC261 backpack vacuum cleaner equipped with Makita 197903-8 2L paper filter bags. The potential PM_10_ loss during sampling caused by decreased filter bags' collection efficiency for smaller fractions was found to be negligible, as the HEPA filter placed after the paper bag in the vacuum cleaner outflow remained visibly clean throughout the entire sampling campaign. Furthermore, scanning electron microscope analysis of samples revealed no reduction in the frequency of small particles (see the Results section).

After sampling, the filter bags were then put in polyethylene bags and transported to laboratory. Because the concentrations only of non-volatile species was determined, and, due to affection of road dust particles by weathering, sunlight and wide temperature range on the road surface before sampling, losses caused by potential degradation during transport and storage of samples were assumed to be negligible. The samples were thus storaged at the room temperature up to 20 °C before their processing.

The preparation of road dust samples for laboratory analyses included the separation of PM_10_ fraction from the raw road dust samples. With respect to health effects, it would be more relevant to use a respirabile fraction (particle with a diameter lower than 5 μm). On the other hand, low small particles abundance in the road dust would make laboratory analysis of the smaller fractions challenging due to limited mass, which negatively affects detection limits. The PM_10_ fraction separation was the compromise between the relevance of health effect and the technical difficulty of the preparation and laboratory processes. The PM_10_ separation was made using low volume sampler LVS6, Sven Leckel – Ingenieurbüro GmbH, Germany, which is routinely used for atmospheric aerosol sampling using 47 mm-diameter-filters. The PM fraction separation in the sampler head is based on particles aerodynamic forces caused by the constant flow through behind the jets of the specific diameter. Large particles, which are not capable to follow the curvature of the air flow behind the jets are impacted on the lubricated plate. In contrast, smaller particles with lower inertia are carried by the air flow further and deposited on the filter, which was subsequently used for laboratory analysis. This standard sampling equipment was modified by utilizing aluminium adapter, which allowed joining vacuum cleaner paper bag to the sampler head. The quality of the PM_10_ separation was verified by the scanning electron microscope, as described below.

### 2.2 Laboratory analyses for the air pollution source apportionment

The determination of the species for air pollution source apportionment in three cities mentioned above was carried out using the PTFE filters (Cytiva Whatman, diameter of 46.2 mm) for gravimetric, elemental, anhydrosacharides, cations, and anions analyses while quarz fibre filters (Ahlstrom-Munksjö, MK5, diameter of 47 mm diameter) were used for organic (OC) and elemental (EC) carbon and PAHs analyses. Prior to the organic and elemental carbon and PAHs sampling, the unloaded quartz fiber filters (QFF) were pre-baked at 800 °C for 3 h.

The Mettler Toledo, XPR6UD5 model was used for gravimetric PM_10_ mass measurement (with a method detection limit of 1.3 μg m^−3^). Clean filters were stabilized at a temperature of 19–21 °C and a relative air humidity of 45–50 % for 48 and 12 h before the first and the second weighing, respectively. The exposed filters were stabilized under the same conditions for 48 and 24 h, respectively.

For the determination of OC and EC concentrations, thermo-optical transmission (Sunset Laboratory Inc., model 4L) adopting the EUSAAR_2 thermal program was used, which is a widely published European standard method for atmospheric aerosol samples [14,15]. A Merck calibration standard (10 μl of saccharose CAS 57-50-1 of carbon concentration 4.2109 μg μl^−1^ dosed on the filter) was used after every ten measured samples to ensure accuracy. The measured OC data were corrected for the field blank values, and the field blanks EC concentration was found to be negligible. The limit of quantification for organic and elemental carbon was determined as three times the concentration of laboratory blanks (3.6 and 0.02 μg m^−3^, respectively).

Anhydrosaccharides and ions (levoglucosan, mannosan, galactosan, SO_4_^2−^, NO_3_^−^, Cl^−^, Br^−^, F^−^, NH_4_^+^, Na^+^, K^+^, Ca^2+^ and Mg^2+^) concentrations were determined with HPAE chromatography (using a two-channel Metrohm, 940 Professional IC Vario with PAD and CD detector for anhydrosugars and ions, respectively). This method has been widely used worldwide for many years [16]. For chromatography analyses, all samples were extracted in 10 mL of pure water (PURELAB® Elga flex) using a horizontal eccentric shaker (350 rotations per minute for 1 h). Water extracts were then filtered through the Cytiva Whatman Puradisc nylon membrane filters (25 mm diameter, 0.45 μm porosity). The Metrohm Metrosep A Supp, Metrosep Carb 2 and Metrosep C6 column guards were used for anhydrosugars, cations and anions, respectively. The limit of quantification and relative uncerainty for individual anhydrosugars were 10 ng m^−3^ and 20 %. In the case of ions these values were 5 ng m^−3^ and 15 %. Ion chromatography is routinely used worldwide for atmospheric aerosol samples analysis in recent source apportionment studies [[17], [18], [19]].

Concentrations of benzo[*a*]pyrene and benzo[g,h,i]perylene were measured using the liquid chromatography method in the Nanotechnology Centre of Technical University of Ostrava with a limit of quantification of 0.15 and 0.33 ng m^−3^, respectively. The measurement relative uncertainty of these species was of 25 %. Since the concentrations of all PAHs in atmospheric aerosol samples usually correlate strongly, they do not significantly enhance the ability to distinguish sources in the PMF model and may introduce a form of duplicity that should be avoided. For this reason, other PAHs were excluded from the source apportionment dataset.

Major and trace elements (Na, Mg, Al, Si, S, K, Ca, Ti, V, Cr, Mn, Fe, Ni, Cu, Zn, As, Se, Cd, In, Sb, Ba, Pb) were measured by ED XRF (Thermo Scientific – ARL Quant X) with the samples autorotated during measurement. The method is well known and has been tested over the long-term for the elemental analyses of aerosol samples [20]. Three measurement repetitions of each sample were averaged to reduce measurement uncertainty. The method calibration was based on the individual Micromatter XRF calibration standard on PTFE filter for each element.

### 2.3 Laboratory analyses of the road dust samples

To assess the quality of the PM_10_ fraction separation from the raw road dust samples, the analysis using scanning electron microscope (SEM) were carried out. For this analysis, the 40 selected road dust samples were prepared in the same way as for other analyses described below. The only difference was use of polycarbonate instead of other filter materials during the preparation. The number of samples for SEM analyses was evenly distributed across seasons and the "A" and "B" site types. Each of the CZ02, CZ03, CZ04, CZ06A, and CZ08A regions was represented by 8 samples.

For the chemical analyses, the PM_10_ fraction of road dust were deposited on the same quarz and PTFE filters as those specified above for the source apportionment. The mass of deposited PM_10_ road dust fraction on filters was gravimetrically determined on the Mettler Toledo scales of the same type as those described above for the source apportionment samples. The quarz filters for the PAHs analyses were pre-baked at 450 °C for 3 h before the PM_10_ deposition.

Major and trace elements (Na, Mg, Al, Si, P, S, Cl, K, Ca, Ti, Mn, Fe, V, Cr, Co, Ni, Cu, Zn, As, Se, Sr, Y, Zr, Mo, Ag, Cd, Sn, Sb, Ba) were measured in the Nanotechnology Centre of Technical University of Ostrava using XEPOS ED XRF (SPECTRO Analytical Instruments, Kleve, Germany). The concentrations of PAHs were measured using the liquid chromatography method in the Nanotechnology Centre of Technical University of Ostrava with a limit of quantification of individual PAHs with four and more aromatic rings between 0.11 and 0.43 ng m^−3^. The measurement relative uncertainty of these non-volatile PAHs was of 25 %.

### 2.4 Data processing and evaluation

Prior to the health risk evaluation, air pollution source apportionment using the Positive Matrix Factorization model (PMF) [21] was carried out in the three cities shown in Fig. 1. The sites included in the model were located within several tens of meters from the roads. The aim of this source apportionment was to determine whether the contribution of road dust resuspension to total traffic PM_10_ emissions is approximately equivalent to its contribution to PM_10_ air concentration. The PMF model has been previously used to distinguish between road dust and contributions from other sources [22]. The model solution was found with 9 meaningful factors. The achieved Q/Qexp ratio was 1.00, with an additional modeling uncertainty of 11 %. The correlation between modeled and observed PM_10_ concentrations was R^2^ = 0.97. The bootstrap test of the model solution showed that more than 95 % of the runs matched all factors. The model was based on 32 strong species and 7 weak species. Since the air pollution source apportionment in this study was only a supporting tool for estimating the resuspended-to-total traffic particles ratio, and it showed good agreement with emission factors (see the Results section), further details of the modeling process are not included here.

The concentrations measured in road dust samples collected from four different site types (city sites distinguished according the traffic intensity to A and B category, industrial and montane background sites) were processed together and the differences among these site types were visualized by box plots in Fig. S6. This approach allowed for clear visibility of differences among them regarding the concentration range and outliers, as well as mean and median values.

The monitoring of vehicle types was not carried out, as the fleet has low spatiotemporal variance in Czech cities. Another reason was that road dust samples represented periods of varying lengths, depending on the time since the last significant precipitation or road cleaning. Attributing the samples to a likely only slightly varying vehicle fleet during such periods would require year-round monitoring across all road profiles, which would result in an extremely high cost-benefit ratio. We did not deal road condition either, as the samples were not assessed individually but statistically processed. The aim was to describe average conditions and their variance at various sites across the Czech Republic, rather than to make conclusions valid only for individual sites.

The main factors influencing the concentrations of heavy metals and PAHs in road dust samples were identified using Pearson correlation cluster analysis and another PMF model. The dataset for these analyses included all measured species in all road dust samples. This PMF model was simplified as the time series was not available for road dust samples (the samples represented rather season than the exact date). PMF was carried out with extra modelling uncertainty of 5 % and DISP analyse showed zero influence of random variations or noise on the base solution. The bootstrap analysis showed a match of factors in only 75 % of the runs. The stability was thus improved by rotating the solution with Fpeak value of +0,5 that led to 100 % matching factors.

The health impacts of metals and PAHs related to road dust resuspension were quantified using the dataset conducted as a part of the yearly air quality reports for the Czech Ministry of Environment by the Czech Hydrometeorological Institute. This dataset consisted of GIS data layer accommodating gridded 1 × 1 km road traffic contributions to the average PM_10_ yearly ambient air concentrations across the Czech Republic. It was generated through chemical transport modeling, which utilized standard European emission factors for all national pollution sources, and was calibrated to measured PM_10_ concentrations at the stations of the National Air Quality Monitoring Network. The details are described in Annex I of the report on air pollution in the Czech Republic in 2021 [23], following the CHMI methodology for data collection and processing [24]. This data layer were multiplied by the average proportion of road dust resuspension in the total traffic PM_10_ contribution. This proportion was determined based on emission factors and PMF source apportionment in three cities shown in Fig. 1. This calculation provided a 1 × 1 km grid representing the contribution of road dust resuspension to PM_10_ air concentrations. The grid was then multiplied by the proportions of PAHs, As, Cd and Ni in PM_10_ road dust fraction. The result was the absolute contribution of road dust resuspension to air concentrations of these pollutants and was used for the subsequent carcinogenic risk assessment. All mentioned spatial calculations were conducted using GRASS GIS.

### 2.5 Health risk estimation

The impact of air pollution on human health depends on ambient air pollution concentration and exposure duration. This evaluation is based on known dose-response relationships derived from epidemiological studies, animal experiments, or studies on health impact in the occupational environments. The result is an estimate of the impact caused by a specific concentration level of the assessed pollutant. To express the level of health risk, an estimate of the occurrence of health effects in exposed individuals is used.

A key indicator of the health impacts of long-term exposure in the presented study was the estimated of the number of premature deaths in the adult population, excluding external causes of death (such an injuries, suicides, etc.). This indicator includes both premature mortality from specific causes of death (such as cardiovascular or respiratory diseases, lung cancer, etc.) and deaths associated with short-term exposure to PM. A commonly used metric is the estimated number of Years of Life Lost (YLLs) due to air pollution with particulate matter. Additional potential indicators are presented in publications by the World Health Organization [25,26]. For the quantitative estimation of health impacts resulting from long-term exposure to suspended particles, the updated global air quality guidelines for Europe [27] were used.

The following indicators of potential health impacts related to road dust resuspension were used:

‒ Estimate of premature mortality
‒ Annual number of hospitalizations at the emergency department for cardiac patients per 100 000 exposed individuals of all ages
‒ Annual rate of emergency respiratory hospitalizations per 100 000 exposed individuals of all ages
‒ Number of days per year using bronchodilators per 1000 children aged 5 to 14
‒ Number of days per year using bronchodilators per 1000 adults aged 20 to 64
‒ Attributable risk of days with respiratory symptoms (LRS) per 1 child aged 5-14
‒ Postneonatal (age 1–12 months) infant mortality, all causes
‒ Prevalence of bronchitis in children aged 6–12 (or 6–18) years
‒ Occurrence of chronic bronchitis in adults (age 18+)
‒ Incidence of asthmatic symptoms in asthmatic children (5–19 years)

The quantification of the mentioned PM_10_ health effects was based on calculating the number of disease cases caused by the increase in PM_10_ concentration due to road dust emissions. The input data for this calculation was the annual average contribution of resuspended road dust to PM_10_ concentrations at the assessed sites.

For the estimation of premature mortality, a baseline of 0 μg m^−3^ was used. For the other indicators mentioned above, a value of 10 μg m^−3^ was not subtracted from the road dust PM_10_ contribution, contrary to the WHO recommendation [25]. The subtraction of 10 μg m^−3^ is relevant only for the total PM_10_ concentration (the sum of road dust and all other sources contributing to PM_10_). Since road dust resuspension represents only a portion of the total PM_10_ concentration, applying such a correction is therefore irrelevant.

In addition to the effect of PM_10_ itself, the carcinogenic health impact of selected pollutants present in road dust was estimated. The probability of new cancer cases due to lifetime exposure (70 years) was calculated for As, Cd, and Ni, as well as for benzo[*a*]pyrene, which represents and includes the carcinogenic effects of other PAHs. As, Cd, and Ni were chosen for the assessment as they are known human carcinogens and are routinely monitored in ambient air by the National Air Quality Monitoring Network of the Czech Republic. As the focus was on the PM_10_ fraction of road dust, with microscopic analyses showing the maximum particle frequency below 5 μm (refer to the Results and discussion), inhalation exposure was considered in the assessment. Oral and dermal exposure were excluded due to the anticipated minimal skin area, and negligible unintentional oral intake, both difficult to quantify. The population carcinogenic risk was calculated by multiplying the population density data published by the Czech Statistical Office with the contributions of metals and benzo[a]pyrene from road dust resuspension. The preparation of the contributions layer is described in Section 2.4. Since the grids of these two datasets are mutually shifted and rotated, and the Czech Statistical Office grid overlaps the Czech Republic border, the GIS analysis produced a slight discrepancy between the actual population of the Czech Republic and the population calculated from all grid squares. The actual population was approximately 10.5 million, based on 2011 data, as the new population census data from 2021 were not yet available. The population sum after the GIS calculations was approximately 10.1 million. This discrepancy was considered insignificant compared to other uncertainties in the risk assessment.

## 3 Results and discussion

### 3.1 The proportion of resuspended road dust in traffic emissions and in the air near roads

The nine factors influencing air quality were identified by the PMF model in selected three cities at the locations, which represent situation within several tens meters from the roads. The contribution of road dust resuspension to the total PM_10_ concentration ranged from 7 to 9 % here, which was from 1.4 to 1.8 μg m^−3^ in absolute terms (average of values for heating and non-heating season). For further details on identified sources please see Figure S1, S2 and S3, and Table S3 in Appendix A (Supplementary data). The portion of resuspended road dust made up from two thirds to three quaters of the total road traffic contribution to atmospheric PM_10_ at surveyed sites. Considering enormous uncertainties of the road dust resuspension quantification, this values are in good agreement with the portion of resuspended particles calculated using emission factors published in U.S. EPA, AP 42 [13] and in EMEP/EEA air pollutant emission inventory guidebook [28]. The calculation utilizing these emission factors showed that the portion of resuspended dust in the total road traffic emissions was of 83 % on the roads adjacent to the mentioned three monitoring sites. Therefore, the relative difference between the PMF model results and the calculation based on these emission factors was less than 20 %. Despite the fact that character of the surveyed sites significantly differred, a relative portion of road dust resuspension in the PM_10_ nearby the roads was similar. This finding allowed for the simplification that road dust was assumed to contribute 75 % to the total traffic emissions in the subsequent evaluation. This compromise value is close to the highest value found by PMF model and somewhat lower than the range calculated using emission factors. Such a contribution is consistent with the statements in previous studies that a most of road dust mass originate from non-exhaust sources in European countries [3,29].

### 3.2 Size distribution, morphology and chemical composition

As a result of the scanning electron microscope analyses, all analyzed samples exhibited lognormal distribution curves, with the highest particle frequency ranging from 1.5 to 5.0 μm. The frequencies of particles with higher aerodynamic diameters than 10 μm were low, indicating effective separation of the PM_10_ fraction and negligible loss of small particles during sample collection and preparation. Microscopic optical properties analyses of road dust samples showed predominantly sharp-edged particles with minimum spherical or rounded particles indicating prevailing mineral origin.

The summary of elemental and PAHs concentrations in the road dust PM_10_ fraction in Table 1 indicates that the median values exceeding 100 mg kg^−1^ decrease in the following order:Fe > Al > Si > K > Na > S > Zn > Mn > Cl > Ca > Ti > P > Mg > Cu > Cr. As the measured concentrations exhibited a lognormal distribution with high extremity for certain elements, the summary statistics, including minimum, quantiles, mean, and maximum, are presented in Table 1 instead of standard deviations. The strongly skewed distribution with pronounced outliers was observed for Na and Cl from major elements, and Cd, Ni, and Sn from trace elements. The lowest relative concentration ranges were found for Al, Fe, and S, as well as for Mo, Y, V, and Zr, respectively.

**Table 1:** Summary of concentrations in road dust PM_10_ (mg kg^−1^).

Species	Minimum	Q25	Median	Mean	Q75	Maximum
BaA	0.20	0.75	1.4	1.9	2.5	9.1
BaP	0.26	0.89	1.7	2.3	3.1	13
BbF	0.25	1.0	1.7	2.5	3.3	23
BghiPRL	0.10	1.0	1.7	2.5	3.2	16
BkF	0.15	0.54	1.0	1.4	1.8	10
CRY	0.33	1.1	1.8	2.5	3.2	20
I123cdP	0.15	0.69	1.4	1.9	2.3	16
Ag	0.08	0.44	0.65	0.80	0.89	9.1
Al	19	11 179	15 020	15 320	19 245	33 247
As	0.39	5.8	8.7	11	15	112
Ba	3.1	26	35	41	48	226
Ca	28	254	348	388	467	1231
Cd	0.08	0.54	0.90	6.0	3.8	199
Cl	40	261	462	2074	1173	45 283
Co	0.15	1.0	5.1	9.1	13	58
Cr	11	91	121	168	173	1226
Cu	3.5	88	129	147	187	585
Fe	2936	23 345	29 948	29 954	36 692	57 962
K	594	4397	6239	6675	8525	16 495
Mg	16	111	199	211	276	908
Mn	85	567	718	824	914	7253
Mo	0.35	48	68	66	84	180
Na	121	1158	2190	4036	3992	51 005
Ni	0.15	1.1	64	151	182	5713
P	13	150	204	219	282	684
S	60	922	1249	1305	1611	3978
Sb	0.08	0.44	0.65	0.80	0.89	9.1
Se	0.12	0.49	0.70	0.93	1.0	9.1
Si	1050	9434	12 646	13 084	17 050	27 587
Sn	0.13	1.8	3.8	6.3	6.6	138
Sr	7.0	55	75	92	99	335
Ti	17	156	217	229	286	916
V	2.9	37	49	51	63	112
Y	0.35	2.6	3.4	3.4	4.2	9.1
Zn	1.2	636	768	834	1023	3644
Zr	0.53	38	54	53	65	174

Mean concentrations of the major components like Al, Fe, Ca, K was approximately 1.5, 3.0, 0.04 and 0.7 percent of PM_10_ mass, respectively, which is a range similar to a previous study carried out in Spain [22], except for Ca, which was observed more than one order of manitude lower (Si was not measured in the cited study).

The absolute concentrations in road dust were published in studies from the two Polish cities, Katowice and Wroclaw (102 samples collected during August 2018, particles <2 mm analyzed), and from Venice, Italy (16 samples in June 2014) [30,31]. The results from Poland are expected to be the most similar to the presented values due to the short distance and similar conditions in this neighboring country to the Czech Republic. The concentration range of Al, Cr, Ni, Zn, and Mn was found to be far wider in the presented research than in the other mentioned studies, likely because the samples were collected under extremely varied conditions, including all seasons, site types, and regions, unlike the other studies. This wide concentration range, which is, for some elements, as high as three orders of magnitude (Al, Cr, Ni, Cd, Sn, Zn), indicates a strong temporal variation of concentrations in road dust, resulting from numerous factors, including weather conditions, road maintenance, and atmospheric deposition of pollution from seasonal sources. The concentration of Mg was one order of magnitude lower in the presented study compared to the Polish results [30] (not determined in the Italian study). Barium concentrations were comparable to the values in the Polish study but lower than those found in Venice. Arsenic concentrations were very low in Italy, while the Polish data fit very well within the range of this study. The Cu concentrations were one order of magnitude higher in the Italian study than in Poland, where they were similar to those in this study.

### 3.3 Spatial variations of the chemical composition

**Fig. 2:**
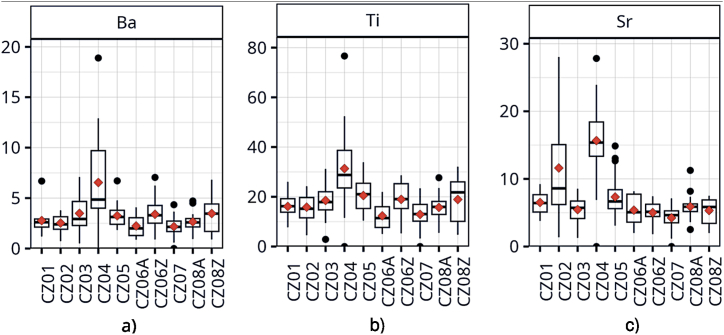
Concentrations in road dust PM_10_ across air quality zones (mg·kg^−1^): a) barium, b) titanium, c) strontium.

Statistical analysis of the laboratory measured concentrations in the road dust samples showed significant regional variation of elemental composition. Fig. 2 shows that the highest concentrations of Ba, Ti, and Sr, which are known as typical crustal species, were found in the north-western part of the Czech Republic (CZ04 Eurostat NUTS-2 Cohesion region), where surface coal mining takes place.

Other findings regarding spatial variations of elemental and PAHs content in road dust are visualized in Supplementary Data (Fig. S4). The raised concentrations of Mn and Se were discovered in the CZ08 region situated in the north-east of the country. They are probably related to emissions from ironworks and steelworks in the cities of Ostrava and Třinec and possibly also to the pollution transport from the Polish part of Silesia where the similar emission sources are located. The Prague and surrounding central Bohemia (CZ01 and CZ02 regions) are distinguished by the relatively highest concentrations of Ca, Cd and As from all Czech regions. Calcium could come from concrete and mortar used for construction works in these most urbanized regions, which are characterized by highest intensity of construction works. Cadmium probably originated from complex city pollution here. In the mentioned CZ01 and CZ02 regions, arsenic originated probably from both residential heating using coal and historical mining activities (some of the sampling sites were in the vicinity of uranium exploatation deposits, which are known for arsenic enrichment). The long-term elevated air concentration of arsenic in the part of the Central Bohemia region, connected to coal-fired boilers in households, was assessed in a previous source apportionment study [32].

The concentrations of PAHs were also regionally specific. The benzo[a]pyrene content in the road dust was the highest in the north part of Moravia and decresed to the south, which is in line with the known air concentrations gradient [33] and possible pollution transport from the Polish part of Silesia [34]. In contrast, the third highest benzo[a]pyrene content within the Czech Republic regions was found in the south Bohemia (CZ03), despite traditionally low ambient air concentrations of the pollutant here [33]. This discrepancy is discused below.

**Fig. 3:**
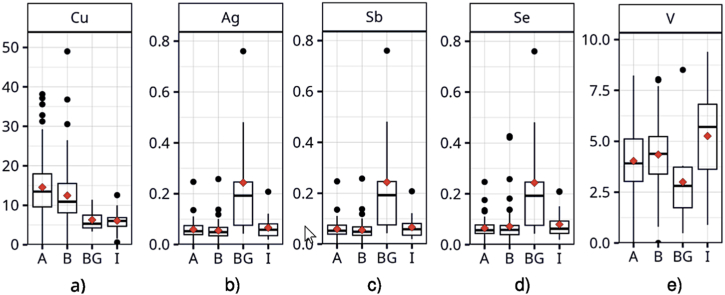
Examples of concentration variances in road dust PM_10_ across different site types (mg·kg^−1^): a) copper, b) silver, c) antimony, d) selenium, e) vanadium; A: roads in cities with daily traffic intensity ≥7500 vehicles; B: roads in cities with daily traffic intensity <7500 vehicles; BG: roads at mountain background sites; I: roads near large industrial facilities.

Road dust elemental composition varied also among the types of sampling sites, while concentrations of PAHs were similar at all types. The variation of all assessed elements and PAHs is depicted in Fig. S6 in Supplementary data. Fig. 3 shows only examples for some of the elements discused below.

The only Cu, and less apparently Zn, reached higher concentration levels on the road types A and B, which represent city roads. The only Cu showed significantly higher concentrations on heavily loaded roads in cities (A-sites) compared to those with lower traffic intensity (B-sites). This finding is in line with the previously discovered higher Cu contamination in road dust in car dense areas [35,36]. Cu and Zn are commonly used among traffic markers in source apportionment studies [[37], [38], [39]]. The presented results support their utilization for such purposes, especially Cu. A further discussion is worth on the Ag, Sb, and Se group. These elements can be attributed to long-range transport because selenium is a marker of industrial coal-burning emissions [40,41], and all three elements are common in coal [[42], [43], [44]]. Interestingly, raised concentrations of these elements in road dust were found in background mountain areas. This is probably due to the cumulative impact of coal-fired power plants located in the Black Triangle, in the border area of Bohemia, Germany, and Poland. The impact of heat and power generation industrial facilities in the North-Bohemian mountain areas was manifested by severe acid rain deforestation in the second half of the 20th century [45]. Due to emission reduction plans, the concentrations of NO_X_ and SO_2_ significantly decreased [23], and acid rains thus faded out, but the deposition of metals still takes place here, as assessed for antimony in a recent study [46]. Results confirm that sites at higher altitudes are more exposed to long-range transport of large combustion plants emissions than lowlands. This thesis is supported by high concentrations of mercury in topsoils of European mountains [47], as mercury is another pollutant originating quite specifically from coal burning in large industrial plants (beside historical and recent mining activities).

### 3.4 Temporal variations of the chemical composition

Most elements and PAHs reached higher concentrations in the colder part of the year (samples collected in winter and spring). The opposite seasonal trend was found for Cu, Zn, Co, Ni, and, to a lesser extent, Ag and Sb (see Fig. S7 in Supplementary data). The seasonal mean values and their standard deviations for the elements with the highest seasonal differences are summarized in Table 2.

**Table 2:** The seasonal mean concentrations and standard deviations in road dust PM_10_ (mg kg^−1^).

Species	spring	summer	autumn	winter
Ag	0.67 ± 0.38	0.91 ± 1.31	0.87 ± 0.84	0.75 ± 0.49
As	19.4 ± 14.8	7.7 ± 4	7.1 ± 7.1	11.2 ± 4.8
Cd	1.5 ± 1.8	9.9 ± 30	7.1 ± 13.9	5.6 ± 10.4
Cl	812 ± 506	401 ± 298	349 ± 323	6994 ± 8740
Cr	175 ± 93	140 ± 133	112 ± 107	250 ± 257
Cu	140 ± 80	169 ± 93	152 ± 101	126 ± 84
Mg	257 ± 126	184 ± 135	160 ± 99	246 ± 139
Na	3274 ± 1968	1938 ± 2201	1582 ± 1196	9636 ± 9968
Ni	8.9 ± 22.2	180 ± 178	279 ± 804	137 ± 160
Sb	0.67 ± 0.38	0.91 ± 1.31	0.87 ± 0.84	0.75 ± 0.49
Zn	766 ± 330	998 ± 380	926 ± 500	637 ± 285
BaP	2.0 ± 2.2	2.3 ± 1.7	2.3 ± 1.9	2.7 ± 2.3

**Fig. 4:**
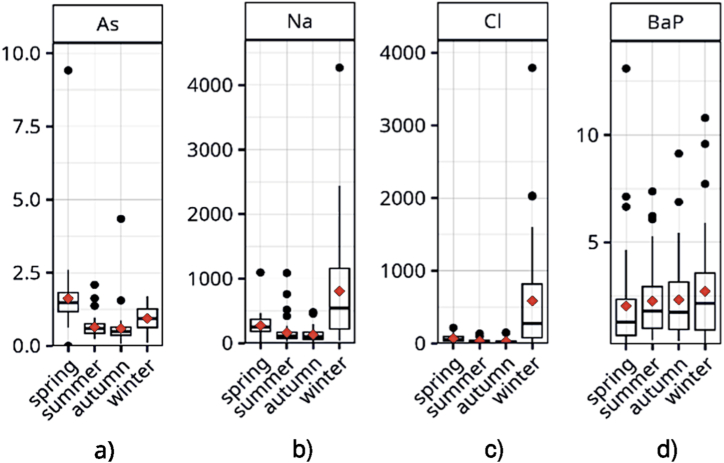
Examples of seasonal trends of concentrations in road dust PM_10_ (mg kg^−1^): a) arsenic, b) sodium, c) chlorine, d) benzo[*a*]pyrene.

As shown in Table 2, concentrations of species commonly attributed to road traffic (Cu, Sb, Zn) and industrial or long-range transported pollution (Ag, Cd, Ni) peaked in road dust during summer and autumn. In contrast, higher concentrations of pollutants characteristic of household heating (As from coal burning and benzo[a]pyrene from both coal and biomass combustion) were observed in the cold season. The highest winter-to-summer concentration ratio was observed for Na and Cl due to the use of winter road salt. Interestingly, in winter and spring, higher concentrations were also observed for typical crustal elements (Si, Al, Fe, Ca, Ba, Ti, Sr), despite wet conditions. This can be explained by the effect of pavements maintenance using gravel and slag in winter. These materials persist in cities until they are removed by rain and road cleaning in the late spring months. Examples of clearly defined seasonal trends are shown in Fig. 4.

### 3.5 Drivers of chemical composition variations

Five clusters of measured species in road dust were identified through correlation analysis: a crustal group (Si, Al, Mg, P, S, Ca, Y, K, Mo, Fe, Ti, Ba, Sr), a cluster of PAHs, a Na-Cl cluster attributed to winter road maintenance, an Ag, Sb, Se, Sn cluster, and a less correlated cluster of Cu-Zn pollution. The crustal group had two sub-groups of strongly correlated elements: Si, Al, Mg, Fe and Ti, Ba, Sr, with the latter being related to surface coal exploitation in the north-western part of the Czech Republic (CZ04 region). For the whole correlation matrix, see Fig. S8 in Supplementary data. The clusters were composed of diagnostic groups indicating not only traffic origin of elements but also the influence of atmospheric deposition. The group of PAHs was probably related to household heating in winter, which is by far the most significant source of PAHs in the Czech Republic [48]. Concentrations of metals in the Ag, Sb, Se, Sn cluster reached the highest concentrations on roads in mountainous areas, indicating the impact of long-range pollution transport from coal-burning power plants, as discussed in Section 3.3. The Cu-Zn cluster was probably connected to traffic wear emissions, as both elements are considered traffic markers [[37], [38], [39]], and the maximum concentrations in road dust were discovered on heavily loaded roads in cities.

By the PMF model, the four factors affecting the road dust chemical composition were identified. The contribution of individual elements and PAHs to these factors is shown in Fig. S9. The “winter” anthropogenic factor was dominated group of crustal elements with road salt, and witer atmospheric deposition (mainly Si, Al, Fe, K, As, Na, Cl). The “year-round” anthropogenic component had the similar composition to winter component but almost without any Al, Na a Cl, and, with higher content of V, Cu a Zn. The “crustal” component which, compared to “winter” anthropogenic component, contained one order of magnitude lower concentrations of metals, S, Si, and, at least one order of magnitude higher crustal elements like Sr, Ba, Ti. The fourth factor was the “PAH” component, which was characterized by a similar content of Al, Fe, K, and Si to the “winter” anthropogenic component, but with the highest concentration of PAHs among all the factors and a significant portion of Se, V, As, Ag, Cr, Sb, and Zn.

The contributions of aforementioned four PMF factors to the total road dust mass in different regions of the country are graphically presented in Fig. S10 in Supplementary data. The highest contribution had the “winter” anthropogenic component, which made up about 60 % of road dust PM_10_ mass. This factor consisted predominantly of crustal species combined with road salt. The second highest portion of the road dust PM_10_ mass consisted of “crustal” component, which was related to deposition of both natural and surface mining particles. In the CZ04 region, which is typical of brown coal surface mining, the portion of the “crustal” component was approximately 40 %, which was twice as high as in other Czech Republic regions. The “year-round” anthropogenic component, which was attributed to road traffic and atmospheric deposition from long-range pollution transport, made up roughly 15 % of the road dust PM_10_ mass, with the exception of samples from the highly traffic-affected part of the CZ06A region, where it accounted for about 40 %. Less important in terms of mass percentage was “PAH” component, which is related likely to deposition of particles originated from coal and biomass-fired boilers in households. The significant impact of atmospheric deposition on PAHs concentrations in road dust was also assessed in previous study [11], where the autors concluded that vehicle emission, coal combustion and petroleum were probably the main contributors of PAHs in road dust. Household heating is long-term the main source of PAHs in the Czech Republic air, as is stated in annually assessed levels and causes of air pollution, national air quality strategies and recent studies [23,24,33]. In the most of Czech Republic regions, content of this component made up first percents of PM_10_ but it took approximately ten percent in the nort-eastern Moravia (CZ08 and CZ08A regions). In some regions, the “PAH” component was thus significant part of road dust in toxicological terms due to high portion of the benzo[a]pyrene and other PAHs with five and more aromatic rings. The decreasing spatial gradient of “PAH” component content from CZ08 to CZ06 region can be explained by atmospheric deposition in accordance with decreasing atmospheric concentrations of benzo[a]pyrene in this corridor [33].

In contrast to the clear link between air quality and PAHs content in road dust in CZ06 to CZ08 regions, no clear explanation was found for slightly raised “PAH” component of road dust in the South Bohemian CZ03 region. The air quality in this region is one of the best across the Czech Republic [23]. There are no specific local PAHs sources in CZ03 region. The atmospheric deposition is thus not expected to be the main reason for the relatively increased PAHs concentrations in the road dust here. The verification and further research of road dust and local air quality focusing PAHs concentrations would thus be appreciated in CZ03 region.

A link between deposition and PAHs content in road dust can relate not only to current air pollution but also to the historical pollution of soils, which subsequently becomes a secondary source of emissions when resuspended. Significant air contamination and increased PAHs loading to soils due to atmospheric deposition in the CZ08 region have continuously taken place for approximately 200 years since heavy industry and residential heating based on coal burning started operations here.

The results presented above show that traffic intensity had only a minor effect on metal and PAHs concentrations. This is clear from the comparison of the concentrations measured in road dust at various sites (see Chapter 3.3 and Fig. S6), where only copper, nickel, and zinc reached higher concentrations on heavily loaded roads. For other elements, no statistically significant difference between differently loaded roads was found. In terms of PM_10_ mass, the most significant elements (Fe, Al, Si, K, Na, S, and Cl) are not related to traffic or are only related to a lesser extent. They originate from soil or other crustal material deposition (Fe, Al, Si, K), road maintenance through salting in winter (Na, Cl), and winter atmospheric deposition of particles from burning processes (As, K, Fe). This claim is consistent with the PMF results, which indicated the largest contribution to the "winter" anthropogenic component and the crustal component, which dominated the PM_10_ mass. In contrast, the year-round component, which includes the traffic chemical fingerprint, accounted for only 15–20 % of the PM_10_ mass. Additionally, seasonal variation also shows dramatic changes in many pollutant concentrations throughout the year. This variation cannot be explained by changes in road traffic, as its seasonal variation is negligible, along with the fact that exhaust and wear emissions vary only slightly over the year. Based on these results, there is no doubt that traffic played only a minor role in the elemental and PAHs concentrations in road dust.

In terms of road dust health effects, particularly As, Cd, Ni, and PAHs variation should be taken into account, as they have strong adverse effects. Considering these species, no statistically significant difference was found between the roads with different traffic intensities. This implies that traffic does not significantly affect their concentrations in road dust, nor the health impact caused by these toxicologically most important components of road dust. Their sources are predominantly other than road traffic. For As and PAHs, residential heating with coal is particularly relevant, as can be seen from their presence in the “winter” and “PAH” PMF components. In contrast, almost no As and PAHs content was discovered in the “year-round” component, which is related to road traffic emissions (see Fig. S9). The differences between site types and the correlation matrix (Figs. S6 and S8) show that the toxicologically extraordinarily harmful Cd and Ni correlate with the industrial polymetallic group (Ag, Se, Sb), suggesting long-range pollution transport, as increased concentrations of these elements in road dust were discovered in montane areas (see Chapter 3.3).

All these findings lead to the conclusion that both the mass concentration of road dust and its health effects are only slightly affected by road traffic, and that they predominantly result from other sources mentioned above.

Spectrum of the only metals that significantly rely on ther traffic intensity (Cu, Ni, Zn) is accordant with other studies, which listed these elements with several others as a part of road dust and traffic wear emissions [6,49,50]. Although many recent studies recommend to utilize various elements as traffic markers in receptor models, presented research showed that copper should be considered as the most reliable in the Czech Republic, as its enrichment in the road dust on heavy-loaded roads was the highest. Concentrations of other elements in road dust depended more significantly on emissions from various other sources and should thus be used as markers of road traffic carefully.

### 3.6 Health impact of road dust resuspension

As described above, a detailed assessment of road dust contribution to PM_10_ concentrations based on roadside measurements and PMF modeling was provided at three sites. These sites represent typical city environments in the Czech Republic, and the data can be extrapolated to most other moderately traffic-loaded city locations in the country.

The estimation of health effects caused by road dust PM_10_ emissions at similar sites in the Czech Republic is presented in Table 3.

**Table 3:** The road dust PM_10_ health effects at monitoring sites.

Indicator	Prague	Ústí n. L.	Zlín
Estimate of premature mortality from resuspension (%)	0.8	1.0	1.1
Annual number of hospitalizations at the emergency department for cardiac patients per 100 000	1	1	1
Annual rate of emergency respiratory hospitalizations per 100 000	1	2	2
Number of days per year using bronchodilators per 1000 children	34	43	49
Number of days per year using bronchodilators per 1000 adults	173	219	246
Attributable risk of days with respiratory symptoms (LRS) per 1 child	2	3	4
Postneonatal infant mortality, all causes (%)	2	2	3
Prevalence of bronchitis in children aged 6–12 years (%)	2	3	3
Occurrence of chronic bronchitis in adults (%)	2	3	3
Incidence of chronic bronchitis in adults (%)	2	2	3

Exposure to the PM_10_ fraction of road dust leads to an increase in premature mortality, postneonatal infant mortality, and the prevalence, occurrence, and incidence of bronchitis by several percent in the Czech Republic. The annual rate of emergency respiratory hospitalizations and the number of days per year using bronchodilators are raised in the range from units to the first hundreds of cases per 1000 people.

Based on the proportion of metals and PAHs in road dust PM_10_ mass, the carcinogenic health impact was estimated. As, Cd, Ni, and benzo[a]pyrene were included in this analysis since, based on their toxicological parameters, they could potentially have the most serious health impact of all the measured species. Table 4 presents the estimation of the probability of new cancer cases due to lifetime exposure (70 years) to these pollutants in resuspended road dust at three assessed monitoring sites.

**Table 4:** The contribution of As, Cd, Ni and PAHs in road dust to carcinogenic risk at measurement sites (unitless).

Site	BaP	As	Cd	Ni
Prague	4.44E-06	8.85E-08	6.52E-08	4.49E-09
Ústí n. L.	4.18E-06	1.04E-07	4.90E-08	1.06E-08
Zlín	6.87E-06	1.29E-07	9.56E-08	1.10E-08

The concentrations of benzo[a]pyrene, As, Cd, and Ni in resuspended road dust, along with the population size, were calculated within the 1 × 1 km square grid covering the entire Czech Republic. These data layers were then used to calculate the population risk excess. Table 5 summarizes key statistics, representing the number of new cancer cases caused by lifetime exposure to the mentioned pollutants in resuspended road dust, calculated for individual 1 × 1 km grid cells.

**Table 5:** The number of new cancer cases caused by lifetime exposure to pollutants in resuspended road dust in 1 × 1 km grid across the Czech Republic (unitless).

Parameter	BaP	As	Cd	Ni
Median	3.9E-08	3.2E-09	3.3E-10	9.8E-09
Mean	2.3E-05	2.6E-06	4.4E-07	9.5E-06
Maximum	1.9E-02	2.5E-03	3.9E-04	1.0E-02

The estimation of the total carcinogenic risk excess for the Czech Republic population (approximately 10 million) caused by exposure to resuspended road dust was 1.8, 0.21, 0.035, and 0.77 for benzo[a]pyrene, As, Cd, and Ni, respectively, and a total of 2.9. Lifetime population exposure to PAHs and heavy metals in road dust thus causes cancer incidence about 3 cases of cancer per ten million inhabitants of the Czech Republic per year above the normal incidence in the population. This result applies under the assumption of lifelong exposure to the assessed concentrations of pollutants.

For context, the text below compares this risk to the total excess cancer incidence caused by the exposure to the total outdoor air concentrations in the country. This estimate is part of the annual evaluation conducted by the National Institute of Public Health based on air quality measurement results from the National Air Quality Monitoring Network of the Czech Republic. In 2023, this estimate ranged from 3 cases per 100 million to 5 cases per 100 thousand inhabitants, depending on the air pollution level of the locality. The overall increase in individual carcinogenic risk for substances with a non-threshold effect (BaP, benzene, Cd, Ni, and As) based on the estimated mean value in urban areas in the Czech Republic not significantly affected by traffic and industry was 7.7E-05, resulting in a population risk of 11 cases per 10 million inhabitants per year. A comparison of these total values with the results presented in this study shows that the average contribution of carcinogenic substances from resuspended road dust accounts for approximately a quarter of cancer cases attributed to total air concentrations. It is worth mentioning that the range of the population risk estimate for minimal and maximal exposure to carcinogenic substances in the air varied from 2.8 to 72.1 additional cases of cancer per year across the Czech Republic. Due to this wide range, the contribution of road dust resuspension to population risk also varies significantly. In low-polluted areas, the inhalation of road dust is likely to contribute even more than the average proportion stated above, while in heavily polluted areas, this proportion may be significantly lower, theoretically constituting only about 5 %.

## 4 Conclusions

The content of crustal species, which made up a majority of the road dust mass, was similar at all sites and regions and variations caused by traffic intensity were suprisingly low, touching only typical elemental traffic markers. Despite these facts, the concentrations of most metals and PAHs in road dust varied significantly both regionally and temporally. As all sampled roads were maintained by salting rather than gritting with inert mineral materials, and because seasonal and regional variations followed seasonal and spatial air pollution trends, the enrichment of metals and PAHs in road dust should be predominantly attributed to deposition from ambient air. Atmospheric deposition thus increased the adverse health effects of traffic through road dust resuspension. This finding, together with the well-known fact that resuspension is a major part of traffic PM_10_ emissions, implies that the future development of propulsion units and brake systems will likely not significantly change the health risks caused by traffic. Therefore, the complex air quality protection measures addressing all important contributors to atmospheric PM_10_ concentrations are required for the effective reduction of deposition rates and, consequently, the health risks associated with road dust resuspension.

Given that a majority of PAHs, arsenic, and certain other metals originate from residential heating in the Czech Republic, the replacement of old coal boilers, ongoing over the past decade, will likely significantly reduce the levels of these pollutants in the air and in road dust due to a decrease in atmospheric deposition rates. Consequently, the risk associated with their resuspension is expected to decrease. Despite these beneficial efforts in the country, there is a problem that hazardous metals in road dust come from various sources, including long-range transported emissions. Therefore, air quality and health problems related to their resuspension by traffic cannot be fully resolved by measures implemented within the Czech Republic. Additionally, the results indicate that the health risk of road dust is related not only to PAHs and metals but also to PM_10_ itself. This suggests that even in the absence of the mentioned pollutants, road dust would still pose a health risk. Therefore, all feasible actions to reduce all components of road dust are necessary, particularly those that make up the majority of particles with a diameter of PM_10_ and smaller, as they can be easily resuspended and are largely respirable. These potential measures should include both prevention, such as reducing atmospheric deposition rates by decreasing air pollution and traffic intensity, and remediation, such as road cleaning and the installation of vegetation barriers along roads to limit the spread of resuspended road dust to a wider area.

## CRediT authorship contribution statement

**Radim Seibert:** Writing – original draft, Project administration, Methodology, Investigation, Data curation, Conceptualization. **Bohumil Kotlík:** Writing – original draft, Supervision, Methodology, Investigation, Formal analysis. **Helena Kazmarová:** Supervision, Methodology, Investigation. **Václav Dombek:** Supervision, Project administration, Methodology, Investigation, Formal analysis. **Vladimíra Volná:** Writing – review & editing, Validation, Supervision. **Daniel Hladký:** Writing – review & editing, Visualization, Validation, Supervision, Data curation, Conceptualization. **Blanka Krejčí:** Writing – review & editing, Supervision.

## Data availability

The data for the analysis were provided by the Czech Hydrometeorological Institute (www.chmi.cz). The assessment is based on measurements at the sites run by the Czech Hydrometeorological Institute.

## Funding

This work was financially supported by 10.13039/100014809Technology Agency of the Czech Republic within the project “Research of PAH and heavy metals atmospheric deposition health effect in connection with the transport induced particles resuspension“, project number SS01010156.

## Declaration of competing interest

The authors declare the following financial interests/personal relationships which may be considered as potential competing interests:Radim Seibert reports financial support was provided by 10.13039/100014809Technology Agency of the Czech Republic. If there are other authors, they declare that they have no known competing financial interests or personal relationships that could have appeared to influence the work reported in this paper.
