# Supplementary material for: Regional and seasonal drivers of metals and PAHs concentrations in road dust and their health implications in the Czech Republic
Source: Heliyon. 2024 Nov 26;10(23):e40725. doi: 10.1016/j.heliyon.2024.e40725 (PMC11650297; doi:10.1016/j.heliyon.2024.e40725)
Supplement: Multimedia component 1 [file mmc1.docx]

**Supplementary data**

Table S1 Number of atmospheric aerosol samples

| Site | Summer | Autumn | Total |
| --- | --- | --- | --- |
| Prague | 60 | 46 | 106 |
| Ústí n. L. | 60 | 60 | 120 |
| Zlín | 57 | 60 | 117 |
| Total | 177 | 166 | 343 |

Table S2 Number of road dust samples

| Site type | Spring | Summer | Autumn | Winter | Total |
| --- | --- | --- | --- | --- | --- |
| A (cities, traffic intensity ≥7500 veh./day) | 21 | 21 | 21 | 21 | 84 |
| B (cities, traffic intensity <7500 veh./day) | 20 | 20 | 20 | 20 | 80 |
| BG (background sites) | 3 | 3 | 2 | 1 | 9 |
| I (industrial) | 6 | 6 | 6 | 6 | 24 |
| Total | 50 | 50 | 49 | 48 | 197 |


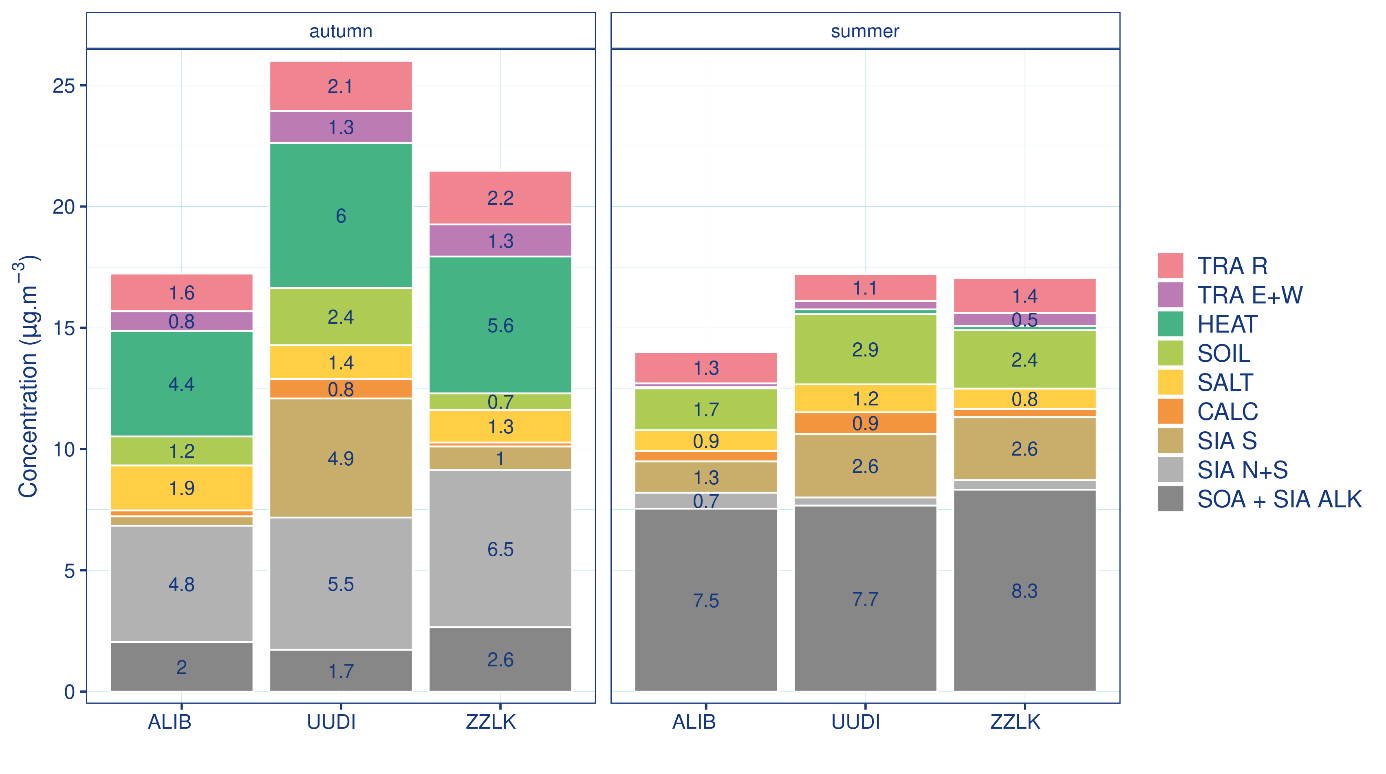


Figure S1 Seasonal PMF contributions to ambient air PM_10_; ALIB: Prague, UUDI: Ústí nad Labem, ZZLK: Zlín; TRA R: road dust resuspension, TRA E+W: traffic exhaust and wear emissions, HEAT: household heating, SOIL: soil and other natural crustal particles, SALT: sea and road salt, CALC: carbonate-rich particles from building reconstruction at the UUDI site, SIA S: ammonium sulphate with As and Se (whole-year type of secondary aerosol), SIA N+S: ammonium nitrate and sulphate (winter type of secondary aerosol), SOA+SIA ALK: carbonaceous particles and sulphates with ammonium, Na, K, Ca and Mg ions, accompanied by Se, Ni, V, Cr (summer complex type of pollution, predominantly secondary organic aerosol)

Table S3 Average PMF contribution to ambient air PM_10_

| Factor | ALIB | | UUDI | | ZZLK | |
| --- | --- | --- | --- | --- | --- | --- |
|  | Absolute | Relative | Absolute | Relative | Absolute | Relative |
|  | [µg.m^-3^] | [-] | [µg.m^-3^] | [-] | [µg.m^-3^] | [-] |
| TRA R | 1.4 | 8.9% | 1.6 | 7.4% | 1.8 | 9.4% |
| TRA E+W | 0.5 | 3.2% | 0.8 | 3.7% | 0.9 | 4.7% |
| HEAT | 2.5 | 15.9% | 3.1 | 14.4% | 2.8 | 14.6% |
| SOIL | 1.4 | 8.9% | 2.6 | 12.0% | 1.6 | 8.3% |
| SALT | 1.4 | 8.9% | 1.3 | 6.0% | 1.1 | 5.7% |
| CALC | 0.3 | 1.9% | 0.8 | 3.7% | 0.3 | 1.6% |
| SIA S | 0.8 | 5.1% | 3.8 | 17.6% | 1.8 | 9.4% |
| SIA N+S | 3 | 19.1% | 2.9 | 13.4% | 3.3 | 17.2% |
| SOA+SIA ALK | 4.4 | 28.0% | 4.7 | 21.8% | 5.6 | 29.2% |
| Total | 15.7 | 100.0% | 21.6 | 100.0% | 19.2 | 100.0% |


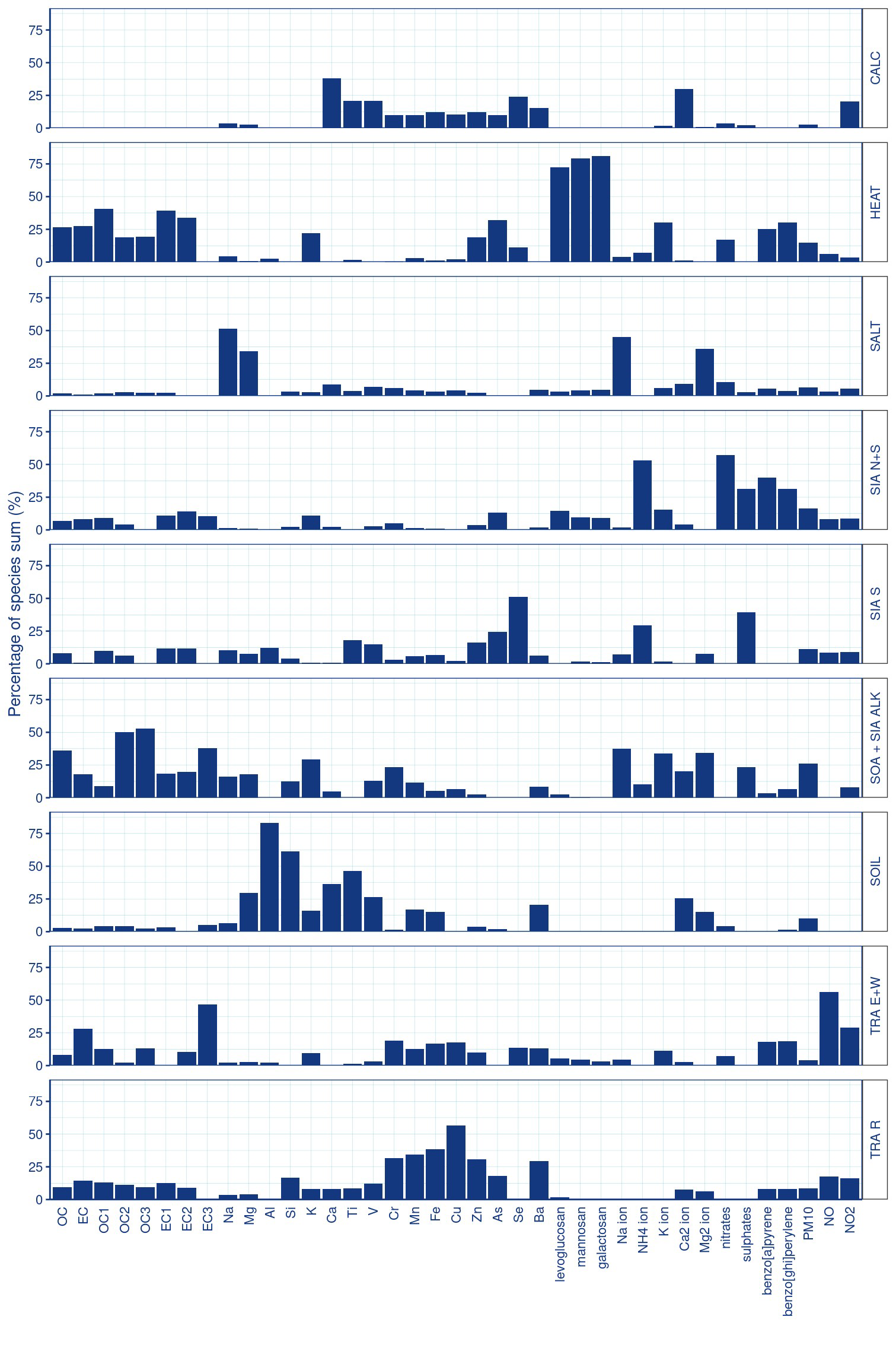


Figure S2 Chemical profiles of ambient air PM_10_ PMF factors at ALIB, UUDI and ZZLK sites.

Figure S3 Time series of ambient air PM_10_ PMF factors at ALIB, UUDI and ZZLK sites


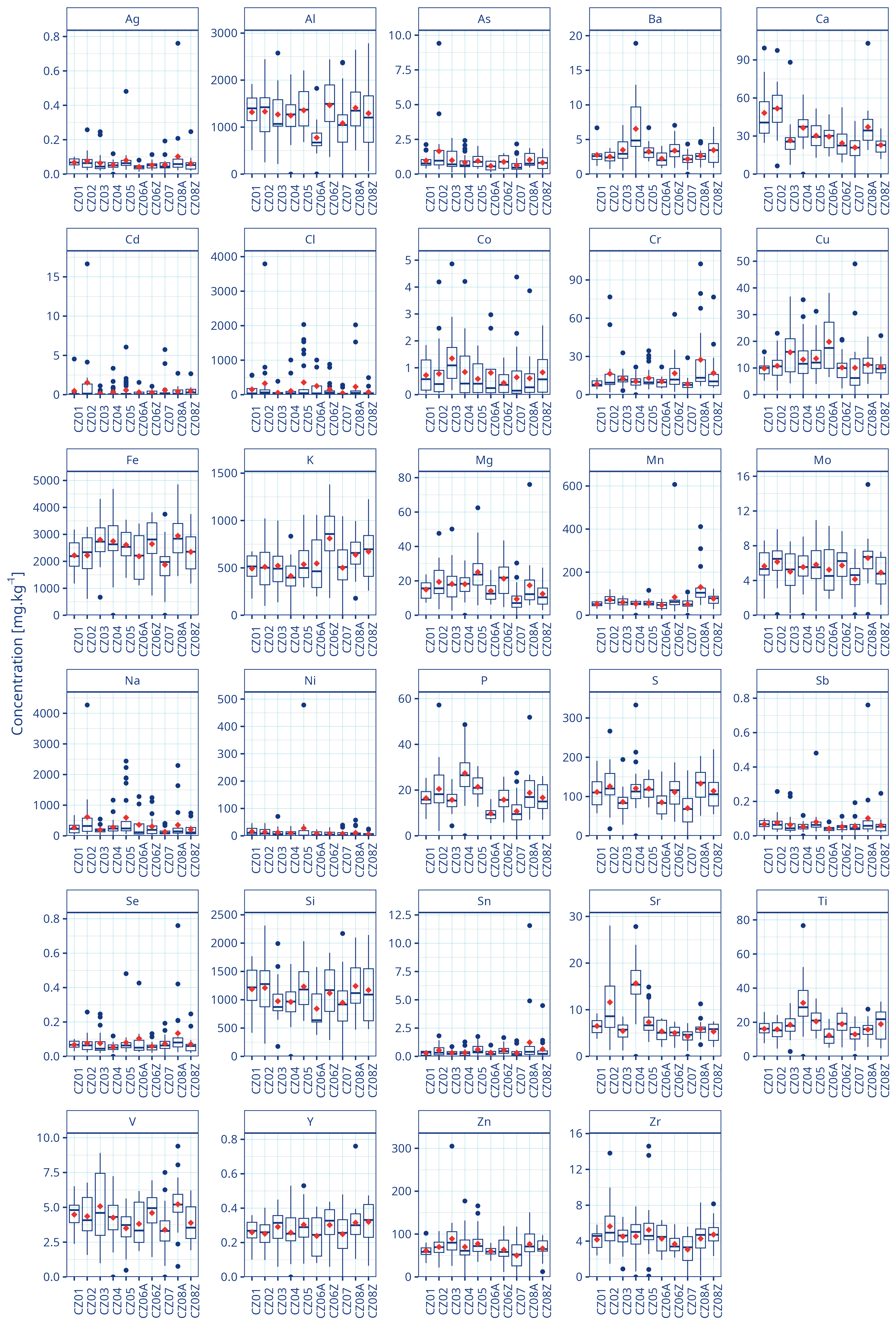


Figure S4 Spatially resolved elemental concentrations in road dust PM_10_


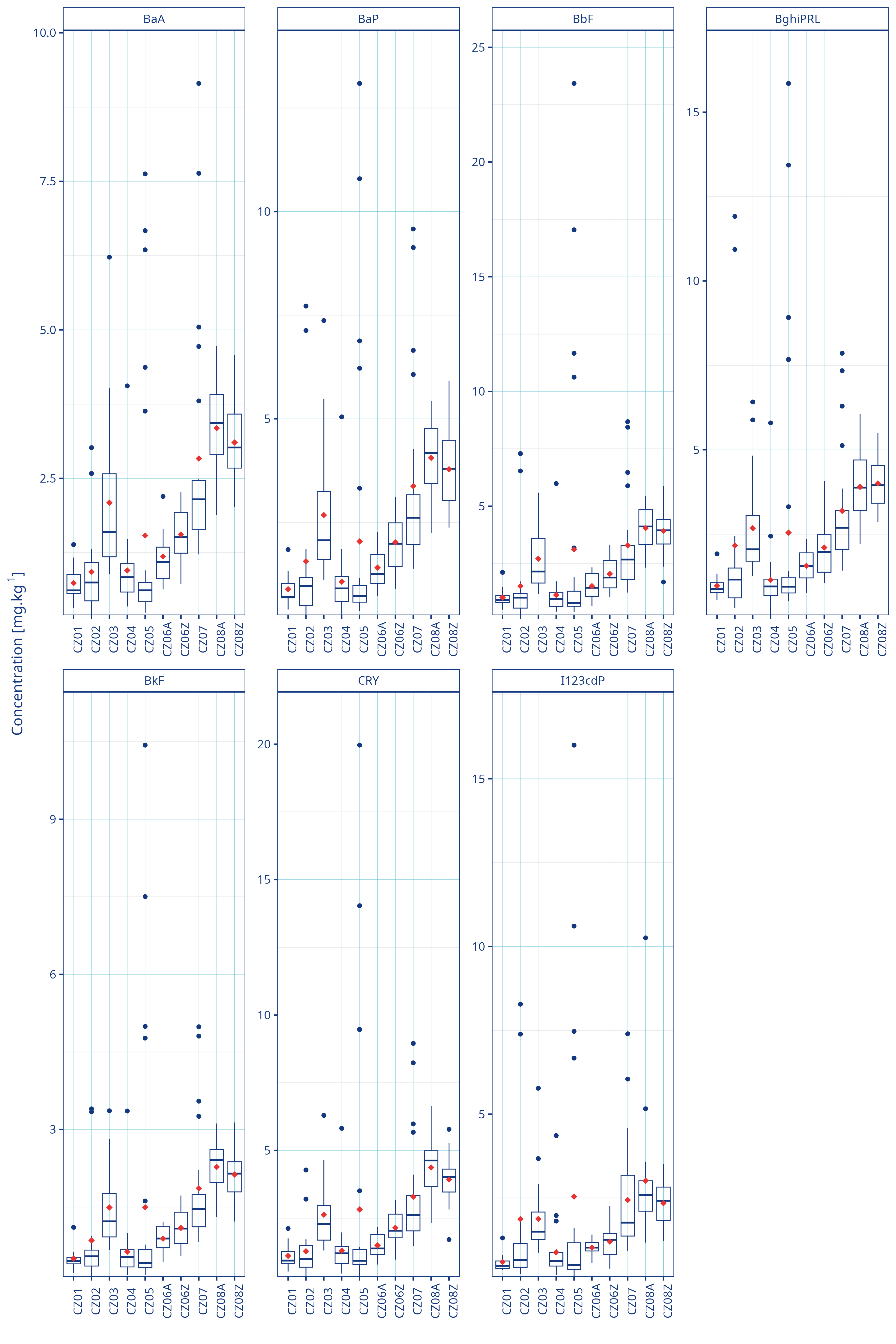


Figure S5 Spatially resolved PAH concentrations in road dust PM_10_


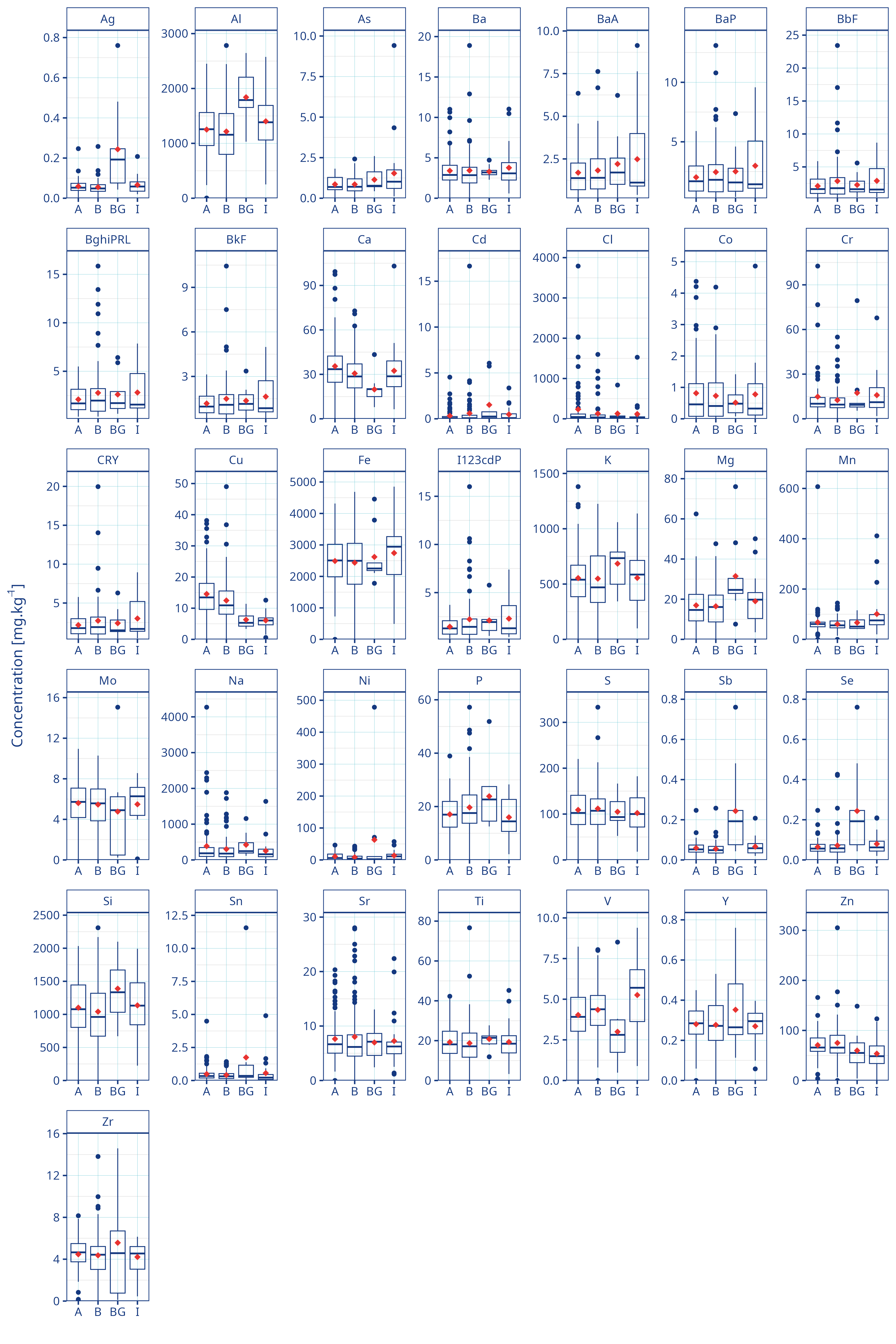


Figure S6 Concentrations in road dust PM_10_ at different location types; A: daily traffic intensity >= 7500 veh., B: daily traffic intensity < 7500 veh., BG: background sites in mountain areas, I: sites near industrial facilities

_
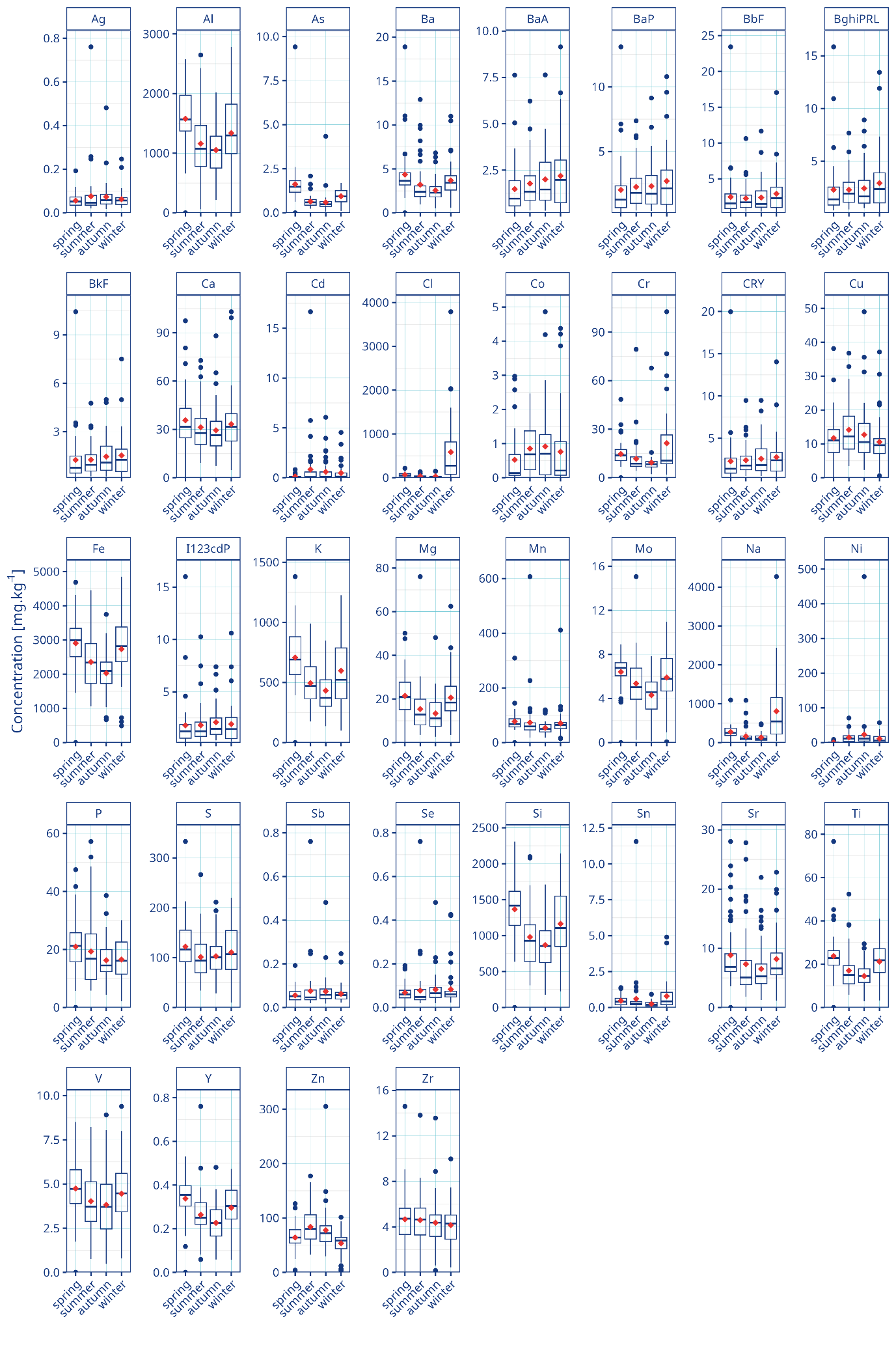
_

Figure S7 Seasonally resolved concentrations in road dust PM_10_


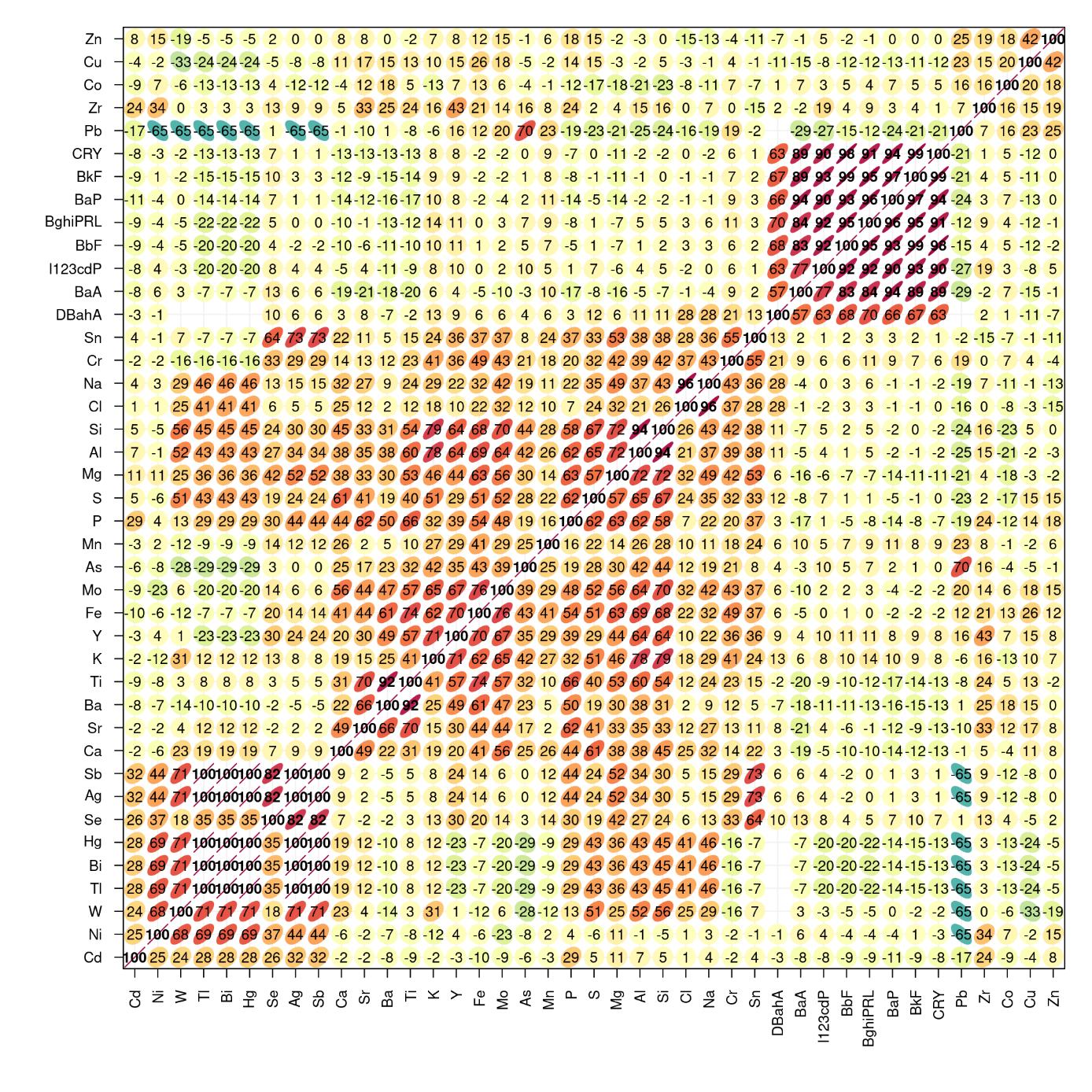
Figure S8 Pearson correlation matrix of elemental concentrations in road dust

Figure S9 The relative elemental and PAH contributions to road dust PMF factors


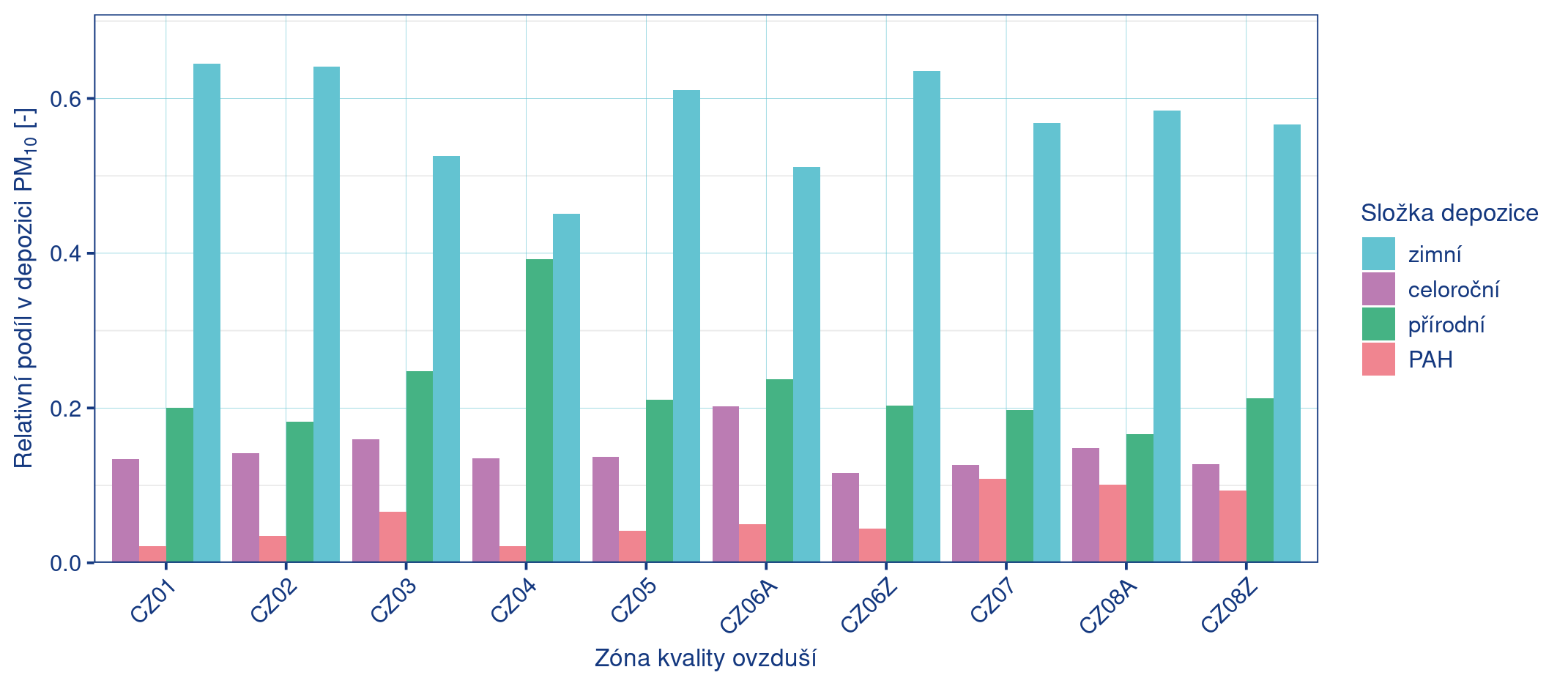


Relative contribution to road dust PM_10_ mass [unitless]

winter

year-round

crustal

PAH

**PMF factor**

Figure S10 Contribution of PMF factors to road dust across the air quality zones
